# Effects of metformin use on aneurysmal subarachnoid hemorrhage outcomes

**DOI:** 10.1007/s00701-025-06516-5

**Published:** 2025-04-10

**Authors:** Angel Bueno, Andrea Becerril-Gaitan, Collins Mokua, Kristina Ramirez-Garcia, Justin Nguyen, Faris Shaker, Tien Nguyen, Antonio Dono, Spiros Blackburn, Peng Roc Chen, Mark Dannenbaum, H. Alex Choi, Arthur L. Day, Jacques J. Morcos, Ching-Jen Chen

**Affiliations:** Vivian L. Smith Department of Neurosurgery, Mcgovern Medical School, The University of Texas Health Science Center at Houston, 6400 Fannin Street, Suite #2800, Houston, TX 77030 USA

**Keywords:** Aneurysmal subarachnoid hemorrhage, Rebleeding, Delayed cerebral ischemia, Cerebral vasospasm, Mortality

## Abstract

**Background:**

Metformin is widely prescribed and has neuroprotective effects in animals, but its impact on brain injury after aneurysmal subarachnoid hemorrhage (aSAH) in humans is unclear.

**Methods:**

This single-center retrospective review assessed patients with aSAH from 2009 to 2023, categorizing them based on pre-admission metformin use. The primary outcome was delayed cerebral ischemia (DCI), while secondary outcomes included in-hospital mortality, rebleeding, angiographic cerebral vasospasm (CVS), and favorable modified Rankin Scale (mRS) scores at discharge and the 3-month follow-up. Outcomes were analyzed using logistic regression. Sensitivity analysis was performed after excluding patients receiving comfort care.

**Results:**

A total of 900 patients were included (47 metformin and 853 non-metformin). DCI rates were similar between groups (38.3% vs. 29.3%, aOR = 1.06 [0.49–2.28]). Rebleeding rates were 4.3% for metformin users and 5.6% for non-users (aOR = 0.47 [0.09–2.51]). In-hospital mortality was 4.3% in metformin users vs. 9.7% in non-users (aOR = 0.47 [0.08–2.84]). Angiographic CVS was 38.3% in metformin users and 52.8% in non-users (aOR = 0.49 [0.23–1.05]), and at 7 days, CVS was 29.8% vs. 47.6% (aOR = 0.46 [0.21–1.01]). Sensitivity analysis showed similar DCI rates (39.1% vs. 30.9%, aOR = 0.98 [0.45–2.15]) but lower CVS at 7 days for metformin users (aOR = 0.44 [0.20–0.98]).

**Conclusion:**

Metformin use before aSAH did not significantly affect the risk of DCI or CVS. However, after excluding comfort care patients, the findings are highly speculative of reduced CVS risk at 7 days post-aSAH. Rebleeding and mortality rates were similar across groups. Future research with larger, multi-institutional datasets is needed to better understand metformin's impact, particularly during and after aSAH.

**Supplementary Information:**

The online version contains supplementary material available at 10.1007/s00701-025-06516-5.

## Introduction

Metformin is one of the most widely prescribed medications globally [8]. Since 2009, it has been endorsed as the first-line oral therapy for type 2 diabetes mellitus (T2DM) by the American Diabetes Association and the European Association for the Study of Diabetes [3]. Owing to its proven efficacy and safety in lowering blood glucose levels by inhibiting hepatic glucose production through both adenosine monophosphate-activated protein kinase (AMPK) dependent and independent mechanisms, metformin has been used in the clinical setting primarily as a diabetes medication [13, 23]. Nonetheless, emerging evidence suggests that metformin may provide additional benefits, including anti-tumor, cardiovascular protective, and anti-inflammatory effects [8, 22, 37].

For instance, metformin has been shown to reduce neuropathological changes and behavioral impairments related to Alzheimer's disease (AD) and Parkinson's disease (PD), indicating that it operates through distinct mechanisms within the central nervous system (CNS) [8, 12]. Furthermore, metformin protects against vascular endothelial injury in pathological conditions such as sepsis and atherosclerosis by reducing inflammation induced by lipopolysaccharides in vascular smooth muscle cells by modulating the peroxisome proliferator-activated receptor gamma (PPARγ) [8, 35]. While numerous studies have clarified the critical pathways involved in regulating vascular endothelial injury, reducing oxidative stress, and enhancing anti-inflammatory effects and mitochondrial function, the role of metformin in aneurysmal subarachnoid hemorrhage (aSAH) outcomes remains unexplored. Thus, the present study aims to reduce this gap by assessing the effect of metformin following aSAH.

## Materials and methods

### Study population

This single-center retrospective study analyzed data from patients with aSAH admitted from January 2009 to March 2023. De-identified demographic and clinical characteristics of included patients were extracted from electronic medical records and compiled for analysis. This study adheres to the guidelines established by the Strengthening the Reporting of Observational Studies in Epidemiology statement (STROBE) [44]. This study was approved by the institutional review board (IRB), and written informed consent was waived because of the retrospective design.

The inclusion criteria for the present study were: (1) 18 years of age or older with a confirmed diagnosis of spontaneous, non-traumatic aSAH, as verified by computed tomography (CT), magnetic resonance imaging (MRI) of the brain, computed tomography angiography (CTA), or digital subtraction angiography (DSA), (2) data on pre-admission use of metformin and, (3) at least 3-months of follow-up. Patients without radiographic evidence of intracranial aneurysm, those with subarachnoid hemorrhage from alternative etiologies, and individuals lost to follow-up were excluded from the study. According to our institutional aSAH treatment protocol, all patients underwent DSA within 24 h of admission and on post-bleed day 7, or as needed if clinical examination or transcranial Doppler (TCD) suggested vasospasm. All patients were admitted to the neurocritical care unit and received management per the guidelines established by the American Heart Association/American Stroke Association [19].

### Baseline characteristics and outcomes measured

The data collected for baseline demographics and clinical characteristics included age, sex, smoking history, alcohol use history, substance abuse history, medication history, past medical history, external ventricular drainage (EVD) placement, World Federation of Neurosurgical Societies (WFNS) grade (1–5), Glasgow Coma Scale (GCS) grade (3–15), Hunt-Hess Scale (HHS) grade (1–5), Intraventricular Hemorrhage (IVH) score (0–23), Modified Fisher Scale grade at admission (1–4), and type of treatment for aSAH (medical management, clipping, or coiling) [5, 15, 16, 18, 32, 34].

Our primary outcome of interest was delayed cerebral ischemia (DCI). Secondary outcomes included the occurrence of rebleeding, in-hospital symptomatic and angiographic cerebral arterial vasospasm (CVS), CVS at 7 days, ventriculoperitoneal shunt (VPS) placement, in-hospital mortality, and patients modified Rankin Scale (mRS) score at discharge and at the 90-day follow-up. Rebleeding was defined as an increase in subarachnoid, intracerebral, or intraventricular hemorrhage on CT or MRI scans accompanied by clinical or neurological deterioration (decrease in GCS) [40, 45]. DCI was defined as focal neurological impairments such as hemiparesis, aphasia, apraxia, hemianopia, neglect, or a decrease of at least 2 points on the GCS [1, 41]. CVS was defined as symptomatic or asymptomatic arterial narrowing on radiological tests, including CTA, magnetic resonance angiography (MRA), or DSA, compared to baseline, with exclusions for atherosclerosis, catheter-induced spasm, or vessel hypoplasia [7, 14, 41]. The mRS scores were categorized into favorable (0–2) and unfavorable (3–6) [6, 11].

### Statistical analysis

Patient baseline characteristics were computed using descriptive and nonparametric statistics. The Chi-square test was used to compare categorical variables among groups. The Mann–Whitney U test was performed to analyze continuous and ordinal numerical variables, comparing medians between groups. Patients were categorized according to their pre-admission use of metformin, with classifications as either users or non-users. Associations between pre-admission use of metformin and the outcomes of interest were assessed using binary logistic regression models. The corresponding odds ratios (ORs) and 95% confidence intervals (CIs) were reported and adjusted with covariates with a *p*-value < 0.05. To avoid listwise deletions due to missing data in the multivariable model, multiple imputation by chained equations with m = 20 was performed. Imputed values for diabetes mellitus (0.77%), WFNS (8.56%), GCS (1.44%) IVH score (9.33%), modified Fisher Scale (4.89%), HHS at admission (0.89%), and mRS at 90-day follow-up (24.89%) were generated by conditional regression models with the following auxiliary variables: age, gender, smoking status, past medical history of hypertension, pre-admission use of metformin, and discharge mRS scores. To further control for confounding, we applied inverse probability of treatment weighting (IPTW) after multiple imputation to adjust for patient baseline covariates. Propensity scores were estimated using a logistic regression model with the following covariates: age, hypertension, diabetes mellitus, coronary artery disease, dyslipidemia, antihypertensive use, statin use, antiplatelet use, and heart failure. Balance among baseline characteristics between cohorts was assessed before and after IPTW. Ors and CIs were then derived from weighted regression models using propensity scores. Statistical significance was defined as *p*-value < 0.05, and all tests were 2-tailed. Furthermore, to address the bias of including comfort care patients (i.e., those designated as comfort care at admission), a sensitivity analysis was performed after their exclusion. All statistical analyses were performed using SPSS (v.29.0.2.0, Humanities & Social Sciences, Stanford University, California, United States of America) and Stata (version 16.1 StataCorp, College Station, Texas) [4].

## Data availability statement

The data generated during this study is available from the corresponding author on a reasonable request. Code availability: Not applicable.

## Results

This institutional study comprised 900 patients diagnosed with aSAH who met the specified inclusion criteria. The median age was 54 years (IQR 45–63). The majority of the patients were females (*n* = 629, 69.1%). Among the entire cohort, 5.2% (*n* = 47) patients were taking metformin before admission.

### Characteristics between groups

When comparing the metformin vs. non-metformin groups (Table 1), a significant age difference was observed, with a median of 59 (IQR 48–68) years among metformin users and 54 years (IQR 44–63) for non-metformin users. Sex and social history were similar. The metformin group had higher rates of hypertension, diabetes mellitus, dyslipidemia, coronary artery disease, and heart failure (*p* = < 0.001, *p* = < 0.001, *p* = < 0.001, *p* = 0.014, *p* = 0.002, respectively). They also used antihypertensives (68% vs. 26.9%, *p* = < 0.001), statins (53.1% vs. 12%, *p* = < 0.001), and antiplatelets (34% vs. 14.6%, *p* = < 0.001) more frequently. Based on modified Fisher and HHS grades at admission, aSAH severity was similar among all groups (*p* = 0.411 and *p* = 0.596, respectively). The GCS scores at admission were comparable between groups, *p* = 0.161. Intraventricular extension of intracerebral hemorrhage was assessed with the IVH score, and no differences were found (*p* = 0.192). EVD placement rates were slightly higher among patients not using metformin (81.5% vs. 76.5%); however, these differences were not statistically significant (*p* = 0.392). The predominant treatment approach for metformin users was endovascular embolization (63.8%) compared to non-users (55.3%), followed by clipping (34% vs. 38.6%) and medical management (2.1% vs. 5.9%), with no statistically significant differences between groups (*p* = 0.373).

**Table 1:** Baseline characteristics

Variable, *n* (%)	All patients (*n* = 900)	Metformin (*n* = 47)	Non-Metformin (*n* = 853)	*p* = value
Age (IQR)	54 (45–63)	59 (48–68)	54 (44–63)	**0.020**
Gender				0.352
Female	629 (69.1)	30 (63.8)	599 (70.2)	
Male	271 (30.1	17 (36.1)	254 (29.7)	
Smoking history	373 (41.4)	20 (42.5)	353 (41.3)	0.874
Alcohol use	334 (37.1)	14 (29.7)	320 (37.5)	0.286
Substance use	100 (11.1)	3 (6.3)	97 (11.3)	0.289
Hypertension	540 (60)	41 (87.2)	499 (58.5)	** < 0.001**
Diabetes mellitus	102 (11.4)	39 (84.8)	63 (7.4)	** < 0.001**
Dyslipidemia	135 (15)	22 (46.8)	113 (13.2)	** < 0.001**
Coronary artery disease	46 (5.1)	6 (12.7)	40 (4.6)	**0.014**
Myocardial infarction	11 (1.2)	1 (2.1)	10 (1.1)	0.562
Ischemic stroke/TIA	25 (2.7)	2 (4.2)	23 (2.6)	0.527
Heart failure	6 (0.6)	2 (4.2)	4 (0.4)	**0.002**
Atrial fibrillation	12 (1.3)	1 (2.1)	11 (1.2)	0.626
Antihypertensive	262 (29.1)	32 (68)	230 (26.9)	** < 0.001**
Statins	128 (14.2)	25 (53.1)	103 (12)	** < 0.001**
Antiplatelets	141 (15.6)	16 (34)	125 (14.6)	** < 0.001**
Glasgow coma scale (IQR)	13 (8–15)	14 (9–15)	13 (7–15)	0.161
Modified Fisher scale on arrival (IQR)	3 (3–3)	3 (3–4)	3 (3–3)	0.411
WFNS on arrival (IQR)	2 (1–4)	2 (1–4)	2 (1–4)	0.250
HHS on arrival (IQR)	3 (2–4)	3 (2–3)	3 (2–4)	0.596
IVH score (IQR)	4 (0–10)	4 (0–8)	4 (0–10)	0.192
EVD	732 (81.3)	36 (76.5)	696 (81.5)	0.392
Treatment of aSAH				0.373
Medical management	52 (5.7)	1 (2.1)	51 (5.9)	
Clipped	346 (38.4)	16 (34)	330 (38.6)	
Coiled	502 (55.7)	30 (63.8)	472 (55.3)	

*IQR* interquartile range, *n* number, % percentage, *OR* odds ratio, *CI* confidence interval, *TIA* transient ischemic attack, *WFNS* World Federation of Neurological Surgeon, *HHS* Hunt and Hess scale, *IVH* Intraventricular hemorrhage, *EVD* External ventricular drain, *aSAH* aneurysmatic subarachnoid hemorrhage

### Outcomes of interest

No statistically significant differences were observed between metformin users and non-metformin users in the rates of DCI (38.3% vs. 29.3%; OR = 1.49 [CI 0.81–2.74]). Similarly, no significant differences were found for rebleeding (4.3% vs. 5.6%; OR = 0.74 [CI 0.17–3.16]), symptomatic CVS (19.1% vs. 21.3%; OR = 0.87 [CI 0.41–1.83]), or angiographic CVS (38.3% vs. 52.8%; OR = 0.55 [CI 0.30–1.01]). At 7-day follow-up with angiography, metformin users had a significantly lower rate and risk of CVS (29.8%) compared to non-users (47.6%), OR = 0.46 (CI 0.24–0.88). In-hospital mortality rates were comparable between groups, with 4.3% in metformin users and 9.7% in non-users, OR = 0.41 (CI 0.09–1.73). The incidence of a favorable mRS score at discharge was similar between groups, with 25.5% in the metformin cohort and 35.3% in the non-metformin group, OR = 0.60 (CI 0.30–1.20). At 90 days, the rates were 61.1% and 57.5%, respectively (OR = 1.10 [CI 0.50–2.30]). Permanent VPS placement rates showed no difference between the groups (23.4% vs. 21.5%), OR = 1.11 (CI 0.55–2.24). After adjusting for potential confounders in the initial model, including age, history of hypertension, diabetes mellitus, dyslipidemia, coronary artery disease, heart failure, antihypertensive use, statin use, and antiplatelet use, the association between metformin use and CVS at the 7-day follow-up was no longer statistically significant (adjusted odds ratio [aOR] = 0.47 [CI 0.21–1.03]). The significance of the remaining outcomes was unaffected. Imputation for missing data did not impact the significance of the previously reported results (Table 2).

**Table 2:** Outcomes

Variable, *n* (%)	Metformin (*n* = 47)	Non-Metformin (*n* = 853)	*Unadjusted OR (95% CI)*	*p* = value	Adjusted OR (95% CI)*	*p* = value	Adjusted OR (95% CI)*^§^	*p* = value
DCI	18 (38.3)	250 (29.3)	1.49 (0.81–2.74)	0.192	1.08 (0.50–2.32)	0.832	1.06 (0.49–2.28)	0.864
Rebleeding	2 (4.3)	48 (5.6)	0.74 (0.17–3.16)	0.690	0.48 (0.09–2.56)	0.543	0.47 (0.09–2.51)	0.383
Symptomatic CVS	9 (19.1)	182 (21.3)	0.87 (0.41–1.83)	0.721	0.91 (0.36–2.29)	0.482	0.90 (0.36–2.27)	0.839
Angiographic CVS	18 (38.3)	450 (52.8)	0.55 (0.30–1.01)	0.056	0.50 (0.24–1.07)	0.077	0.49 (0.23–1.05)	0.068
CVS at 7-days Follow-up	14 (29.8)	406 (47.6)	0.46 (0.24–0.88)	**0.020**	0.47 (0.21–1.03)	0.060	0.46 (0.21–1.01)	0.055
Permanent VPS	11 (23.4)	183 (21.5)	1.11 (0.55–2.24)	0.752	0.72 (0.31–1.69)	0.462	0.72 (0.31–1.66)	0.444
In-hospital mortality	2 (4.3)	83 (9.7)	0.41 (0.09–1.73)	0.226	0.48 (0.08–2.87)	0.424	0.47 (0.08–2.84)	0.417
Favorable mRS at discharge†	12 (25.5)	301 (35.3)	0.60 (0.30–1.20)	0.175	1.48 (0.62–3.52)	0.372	1.45 (0.61–3.45)	0.394
Favorable mRS at 90 days†	22 (61.1)	368 (57.5)	1.10 (0.50–2.30)	0.670	1.96 (0.80–4.77)	0.136	1.70 (0.69–4.15)	0.243

*IQR* interquartile range, *n* number, % percentage, *OR* odds ratio, *CI* confidence interval, *DCI* Delayed cerebral ischemia, *CVS*, Cerebral arterial vasospasm, *VPS* ventriculoperitoneal shunt, *mRS* Modified Rankin Score, †Favoral mRS: 0–2; *Adjusted for age, hypertension, diabetes mellitus, dyslipidemia, coronary artery disease, antihypertensive, statins, antiplatelets and heart failure; ^***§***^Values determined following multiple imputation by chained equations with *m* = 20

### Inverse probability of treatment weighting analysis

The detailed covariates and outcomes of interest following IPTW analysis are presented in Supplementary Tables 1 and 2. DCI rates were similar between metformin and non-metformin users, OR = 0.69 [CI 0.26–1.79]). Patients with pre-admission metformin use had a lower likelihood of developing angiographic CVS during hospitalization after aSAH than those without metformin use, OR = 0.25 (CI 0.10–0.65). CVS rates at the 7-day follow-up were comparable between groups (29.8% vs. 47.6%), OR = 0.37 (CI 0.13–1.01). No significant associations were found in the remaining outcomes of interest.

### Sensitivity analysis

Baseline characteristics and outcomes of interest after excluding comfort care patients (*n* = 52) are shown in Tables 3 and 4. DCI rates were comparable between groups, with 39.1% in metformin users and 30.9% in non-metformin users (OR = 1.43 [CI 0.77–2.64]). Patients with metformin use before admission had a lower likelihood of developing angiographic CVS during hospitalization after aSAH than those without metformin use (39.1% vs. 55.9%), OR = 0.50 (CI 0.27–0.93). After adjusting for potential confounders, the difference was non-significant (aOR = 0.47 [CI 0.22–1.02]). However, after performing multiple imputation, the initial statistical significance was maintained (aOR = 0.46 [CI 0.21–0.99]. CVS at the 7-day follow-up was significantly different between groups, with a lower rate observed in the metformin group (13% vs. 24.2%), OR = 0.43 (CI 0.22–0.81). Following adjustment for potential confounders, the difference was non-significant (aOR = 0.45 [CI 0.20–1.00]). Nevertheless, after performing multiple imputation, the initial statistical significance was maintained (aOR = 0.44 [CI 0.20–0.98]. No significant changes were observed in the remaining outcomes of interest.

**Table 3:** Baseline characteristics after excluding patients who did not undergo intervention for underlying aneurysm

Variable, *n* (%)	All patients (*n* = 848)	Metformin (*n* = 46)	Non-Metformin (*n* = 802)	*p* = value
Age (IQR)	53 (44–63)	59 (48–68)	53 (44–62)	**0.012**
Gender				
Female	585 (69)	29 (63)	556 (69.3)	0.370
Male	263 (31)	17 (37)	246 (30.6)	
Smoking history	356 (42)	19 (41.3)	337 (42)	0.924
Alcohol use	325 (38.3)	14 (30.4)	311 (38.7)	0.258
Substance use	98 (11.5)	3 (6.5)	95 (11.8)	0.272
Hypertension	508 (60)	40 (87)	468 (58.3)	** < 0.001**
Diabetes mellitus	99 (11.8)	38 (84.4)	61 (7.7)	** < 0.001**
Dyslipidemia	125 (14.7)	22 (47.8)	103 (12.8)	** < 0.001**
Coronary artery disease	36 (4.2)	6 (13)	34 (4.2)	**0.006**
Myocardial infarction	10 (1.2)	1 (2.1)	9 (1.1)	0.520
Ischemic stroke/TIA	23 (2.7)	2 (4.3)	21 (2.6)	0.483
Heart failure	4 (0.4)	2 (4.3)	2 (0.2)	** < 0.001**
Atrial fibrillation	11 (1.3)	1 (2.1)	10 (1.2)	0.589
Antihypertensive	246 (29)	31 (67.4)	215 (27)	** < 0.001**
Statins	118 (14)	25 (54.3)	93 (11.5)	** < 0.001**
Antiplatelets	135 (16)	16 (34.7)	119 (14.8)	** < 0.001**
Glasgow coma scale (IQR)	13 (8–15)	14 (9.75–15)	13 (8–15)	0.203
Modified Fisher scale on arrival (IQR)	3 (3–3)	3 (3–4)	3 (3–3)	0.355
WFNS on arrival (IQR)	2 (1–4)	1.5 (1–3.75)	2 (1–4)	0.343
HHS on arrival (IQR)	3 (2–3)	3 (2–3)	3 (2–3)	0.809
IVH score (IQR)	4 (0.0–10)	4 (0.0–7.25)	4 (0.0–10)	0.190
EVD	704 (83)	35 (76)	669 (83.4)	0.198
Treatment of aSAH				0.393
Clipped	346 (41)	16 (34.7)	330 (41.1)	
Coiled	502 (59)	30 (65.2)	472 (58.8)	

*IQR* interquartile range, *n* number, % percentage, *OR* odds ratio, *CI* confidence interval, *TIA* transient ischemic attack, *WFNS* World Federation of Neurological Surgeon, *HHS* Hunt and Hess scale, *IVH* Intraventricular hemorrhage, *EVD* External ventricular drain, *aSAH* aneurysmatic subarachnoid hemorrhage

**Table 4:** Outcomes of interest after excluding comfort care patients

Variable, *n* (%)	Metformin (*n* = 46)	Non-Metformin (*n* = 802)	*Unadjusted OR (95% CI)*	*p* = value	Adjusted OR (95% CI)*	*p* = value	Adjusted OR (95% CI)*^§^	*p* = value
DCI	18 (39.1)	248 (30.9)	1.43 (0.77–2.64)	0.245	1.00 (0.46–2.20)	0.983	0.98 (0.45–2.15)	0.979
Rebleeding	2 (4.3)	43 (5.4)	0.80 (0.18–3.42)	0.766	0.41 (0.07–2.29)	0.314	0.40 (0.07–2.23)	0.301
Symptomatic CVS	9 (19.6)	181 (22.6)	0.83 (0.39–1.76)	0.635	0.86 (0.34–2.19)	0.759	0.85 (0.33–2.16)	0.746
Angiographic CVS	18 (39.1)	448 (55.9)	0.50 (0.27–0.93)	**0.029**	0.47 (0.22–1.02)	0.057	0.46 (0.21–0.99)	**0.050**
CVS at 7-days Follow-up	6 (13.0)	194 (24.2)	0.43 (0.22–0.81)	**0.010**	0.45 (0.20–1.00)	0.052	0.44 (0.20–0.98)	**0.047**
Permanent VPS	11 (23.9)	182 (22.7)	1.07 (0.53–2.15)	0.848	0.72 (0.30–1.67)	0.448	0.71 (0.30–1.65)	0.427
In-hospital mortality	1 (2.2)	42 (5.2)	0.40 (0.05–2.98)	0.373	0.30 (0.02–3.88)	0.363	0.30 (0.02–3.85)	0.360
Favorable mRS at discharge†	12 (26.1)	296 (36.9)	1.04 (0.51–2.11)	0.903	1.47 (0.62–3.52)	0.377	1.45 (0.61–3.44)	0.397
Favorable mRS at 90 days†	22 (47.8)	366 (45.6)	0.60 (0.30–1.18)	0.141	2.06 (0.83–5.09)	0.117	1.71 (0.69–4.24)	0.241

*IQR* interquartile range, *n* number, % percentage, *OR* odds ratio, *CI* confidence interval, *DCI* Delayed cerebral ischemia, *CVS* Cerebral arterial vasospasm, *VPS* ventriculoperitoneal shunt, *mRS* Modified Rankin Score, †Favoral mRS: 0–2; *Adjusted for age, hypertension, diabetes mellitus, dyslipidemia, coronary artery disease, antihypertensive, statins, antiplatelets and heart failure; ^***§***^Values determined following multiple imputation by chained equations with *m* = 20

## Discussion

This retrospective review found no significant difference in DCI between metformin and non-metformin users. Metformin users had a 56% lower odds of CVS at the 7-day follow-up (13% vs. 24.2%) after excluding comfort care patients. In-hospital mortality was lower for metformin users (4.3% vs. 9.7%), although not statistically significant. Functional outcomes at discharge showed no differences between groups. Overall, the primary outcome, DCI, and most secondary outcomes were not significantly different among groups.

The key to further improving morbidity and mortality after aSAH requires the identification and mitigation of factors or events that exert a positive or negative influence on patient outcomes [39]. The relatively low-risk profile and high effectiveness of metformin have led to increased efforts to expand its influence on CNS pathologies, highlighting the importance and feasibility of this study. Metformin has been shown to have beneficial effects on neurodegenerative diseases [28]. T2DM and peripheral insulin resistance increase the risk of AD by 50–100% due to the development of brain insulin resistance and inhibition of neuronal insulin receptors [9]. In studies, metformin has been found to reduce amyloid beta (Aβ) levels in mice with T2DM and enhance the activity of insulin-degrading enzyme and neprilysin, which are both involved in the degradation of insulin and Aβ in both the hippocampus and cerebral cortex [2].

For instance, a preclinical trial by Zhao et al. suggested that AMPK pathway activation played a critical role in metformin-induced neuronal protection against reactive oxygen species [46]. It has been demonstrated that following aSAH, endothelial damage induces early inflammation characterized by endothelin production by astrocytes and increased endothelial cells [31, 33, 36]. This inflammation, combined with reduced cerebrospinal fluid flow and the restoration of endothelial tight junctions, leads to the accumulation of neutrophils and macrophages in the subarachnoid space, resulting in the release of oxidative radicals and endothelins that may cause vasoconstriction, cerebritis, and chemical meningitis [43]. These latest observations correspond with the reduced risk of angiographic vasospasm observed in our metformin user group at the 7-day follow-up. In a preclinical trial, Liu et al. demonstrated that metformin, via an AMPK pathway, induced down-regulation of intercellular adhesion molecule- 1, which reduced neutrophil infiltration and improved inflammatory responses following ischemic injury [20]. Although our results indicated no statistically significant difference in DCI rates between groups, CVS was observed to be lower in the metformin group. Metformin may preferentially affect large vessels without significantly affecting small vessels, potentially explaining the lack of impact on DCI. Whereas it is known that CVS does not always lead to DCI and does not consistently correlate with clinical outcomes, severe vasospasm is associated with a higher incidence of DCI [10, 24, 26, 27]. While not a reliable surrogate for clinical outcomes, CVS remains the only modifiable indicator for DCI [25].

Mortality rates after aSAH vary between 22 and 50% in current studies [17, 21, 29, 30, 38, 42]. Our study indicated that metformin users exhibited a non-statistically significant lower mortality rate (4.3% vs. 9.7%) compared to non-users. Recent research by Li et al. demonstrated that administering metformin in rat models resulted in a decreased incidence and rupture rate of intracranial aneurysms, accompanied by upregulation of AMPK, α-smooth muscle actin, and SM22α, while treatment with the AMPK antagonist compound C produced opposing effects. Additional trials have demonstrated that metformin significantly enhances cognitive functions following controlled cortical impact injury in mice, showing improved spatial learning and nest-building behaviors, while injured mice exhibited increased ramification of microglial processes, indicating reduced neuroinflammation [12]. However, further research would be needed to evaluate the effect of metformin on morbidity and in-hospital mortality rates.

## Limitations

The present study has several limitations that should be acknowledged. First, the data were collected from a single tertiary-care center, which may limit the generalizability of our findings. Future research should involve multi-institutional datasets to enhance the external validity of the results. The retrospective and observational nature of the study introduces potential residual confounding, affecting our ability to fully elucidate the relationship between metformin use and outcomes in aSAH. Additionally, selection bias is a concern, compounded by the relatively small cohort of aSAH patients receiving metformin at our institution. A further limitation is that metformin was taken before aSAH, rather than during the aSAH event, as per our institution’s protocol, patients with aSAH are placed on insulin scales rather than restarting antidiabetic drugs to avoid complications in this critical state. The lack of data on the duration of metformin treatment for most patients further restricts the depth of our analysis. Despite these limitations, our study contributes valuable preliminary insights into the effect of pre-admission metformin use on aSAH outcomes, thereby addressing a gap in current understanding.

## Conclusions

Metformin use before aSAH did not appear to reduce the risk of DCI or CVS. Functional outcomes and mortality rates were also not significantly different with metformin use. In aSAH patients who were not designated comfort care, prior metformin use seemed to decrease the risk of CVS 7 days after aSAH. The significance of this finding is unclear, as any effects of metformin stemmed from pre-aSAH use and the relationship between CVS and DCI remains equivocal.

## Supplementary Information

Below is the link to the electronic supplementary material.

Supplementary file1 (DOCX 18 KB)

## Abbreviations

aSAH: Aneurysmal Subarachnoid hemorrhage
DCI: Delayed cerebral ischemia
CVS: Cerebral vasospasm
AMPK: Adenosine monophosphate-activated protein kinase
PPARγ: Peroxisome proliferator-activated receptor gamma
T2DM: Type 2 diabetes mellitus
mRS: Modified Rankin scale
GCS: Glasgow coma scale
HHS: Hunt-Hess scale
CT: Computed tomography
MRI: Magnetic resonance imaging
TCD: Transcranial Doppler
EVD: External ventricular drainage
VPS: Ventriculoperitoneal shunt
CTA: Computed tomography angiography
DSA: Digital subtraction angiography
OR: Odds ratio
