# Supplementary material for: Effects of metformin use on aneurysmal subarachnoid hemorrhage outcomes
Source: Acta Neurochir (Wien). 2025 Apr 10;167(1):103. doi: 10.1007/s00701-025-06516-5 (PMC11985594; doi:10.1007/s00701-025-06516-5)
Supplement: Supplementary file 1 — Supplementary file1 (DOCX 18 KB) [file 701_2025_6516_MOESM1_ESM.docx]

**Supplementary Table 1.** Outcomes of interest following inverse probability of treatment weighting after multiple imputation on the entire cohort (n=900)

| **Variable, n (%)** | **Metformin (n=47)** | **Non-Metformin (n=853)** | ***OR after IPWT (95% CI)**** | ***p*=value** |
| --- | --- | --- | --- | --- |
| **DCI** | 18 (38.3) | 250 (29.3) | 0.69 (0.26-1.79) | 0.453 |
| **Rebleeding** | 2 (4.3) | 48 (5.6) | 0.44 (0.16-1.22) | 0.118 |
| **Symptomatic CVS** | 9 (19.1) | 182 (21.3) | 0.70 (0.22-2.18) | 0.542 |
| **Angiographic CVS** | 18 (38.3) | 450 (52.8) | 0.25 (0.10-0.65) | **0.004** |
| **CVS at 7-days Follow-up** | 14 (29.8) | 406 (47.6) | 0.37 (0.13-1.01) | 0.053 |
| **Permanent VPS** | 11 (23.4) | 183 (21.5) | 0.82 (0.29-2.33) | 0.718 |
| **In-hospital mortality** | 2 (4.3) | 83 (9.7) | 0.51 (0.06-4.00) | 0.530 |
| **Favorable mRS at discharge**† | 12 (25.5) | 301 (35.3) | 1.92 (0.65-5.68) | 0.233 |
| **Favorable mRS at 90 days**† | 22 (61.1) | 368 (57.5) | 1.80 (0.62-5.17) | 0.272 |
| IQR: interquartile range; n: number; %: percentage; OR: odds ratio; CI: confidence interval; IPTW: inverse probability of treatment weighting; DCI: Delayed cerebral ischemia; CVS: Cerebral arterial vasospasm; VPS: ventriculoperitoneal shunt; mRS: Modified Rankin Score; †Favoral mRS: 0-2. *Adjusted for age, hypertension, diabetes mellitus, dyslipidemia, coronary artery disease, antihypertensive, statins, antiplatelets and heart failure. | | | | |

**Supplementary Table 2.** Balance diagnostics using standardized mean differences before and after inverse probability of treatment weighting with propensity scores on the entire cohort (n=900)

| **Variable** | **Mean value before IPWT** | | **Unadjusted SMD** | **Mean value after IPWT** | | **Adjusted SMD** |
| --- | --- | --- | --- | --- | --- | --- |
|  | **Metformin** | **Non-Metformin** |  | **Metformin** | **Non-Metformin** |  |
| Age | 58.51 | 53.71 | 0.3744 | 57.25 | 53.98 | 0.2547 |
| Hypertension | 0.87 | 0.58 | 0.6802 | 0.74 | 0.60 | 0.3170 |
| Diabetes mellitus | 0.84 | 0.07 | 0.3540 | 0.10 | 0.05 | 0.2250 |
| Dyslipidemia | 0.46 | 0.13 | 0.7808 | 0.17 | 0.15 | 0.0686 |
| Coronary artery disease | 0.12 | 0.04 | 0.2868 | 0.06 | 0.05 | 0.0583 |
| Antihypertensive | 0.68 | 0.26 | 0.8982 | 0.37 | 0.29 | 0.1672 |
| Statins | 0.53 | 0.12 | 0.9681 | 0.20 | 0.14 | 0.1566 |
| Antiplatelets | 0.34 | 0.14 | 0.4604 | 0.19 | 0.15 | 0.1113 |
| Heart failure | 0.04 | 0.00 | 0.2488 | 0.00 | 0.00 | -0.0107 |
| IPWT: inverse probability of treatment weighting; SMD: standardized mean difference. | | | | | | |
